# Social impacts of corruption upon community resilience and poverty

**DOI:** 10.4102/jamba.v9i1.391

**Published:** 2017-05-26

**Authors:** James Lewis

**Affiliations:** 1 Datum International, South Gloucestershire, United Kingdom

## Abstract

Corruption at all levels of all societies is a behavioural consequence of power and greed. With no rulebook, corruption is covert, opportunistic, repetitive and powerful, reliant upon dominance, fear and unspoken codes: a significant component of the ‘quiet violence’. Descriptions of financial corruption in China, Italy and Africa lead into a discussion of ‘grand’, ‘political’ and ‘petty’ corruption. Social consequences are given emphasis but elude analysis; those in Bangladesh and the Philippines are considered against prerequisites for resilience. People most dependent upon self-reliance are most prone to its erosion by exploitation, ubiquitous impediments to prerequisites of resilience – latent abilities to ‘accommodate and recover’ and to ‘change in order to survive’. Rarely spoken of to those it does not dominate, for long-term effectiveness, sustainability and reliability, eradication of corrupt practices should be prerequisite to initiatives for climate change, poverty reduction, disaster risk reduction and resilience.

## Résumé

Corruption , existing at all levels of all societies in varying degrees, is a behavioural consequence of power and greed in contexts of inadequate governance. With no published rulebook or formula with which to comply, corruption is covert, repetitively opportunistic and powerfully reliant upon dominance and fear within unwritten and unspoken codes. It is therefore an understatement that, consequently, corrupt practices do not readily lend themselves to scientific analysis. Instead, investigation of its consequences amongst the poor has to be necessarily ad hoc and gathered from relatively few published sources which have become available over time. For the purposes of this assessment of its social impacts upon resilience and poverty, extracts have been gathered of its variety of methods and pervasive consequences; as with corruption itself, its procedures are evasive and do not readily lend themselves to formal research.

Literature on the social impacts of corruption is limited, a definitive analysis of corruption and its social consequences being not, as yet, a practicable undertaking. This short contribution reflects some preliminary investigation of the social impacts of corrupt practices upon the poorer sectors of societies, where and when accessible literature has ensued.

Corrupt practices amongst high level political, commercial and industrial dealings, rightly receiving media attention, may become the commencement of long-term trickle-down consequences for the poor which, at society’s lower levels, are unlikely to attract either scientific or media notice. Whilst the scale of corruption on China, Italy and Africa here receive mention, the impacts of corruption upon the poor of these and other societies in Africa, Bangladesh and the Philippines, for example, reveal social consequences which are here examined and considered against required prerequisites for resilience. Those societies and communities most reliant upon their own resilience to crises of any kind are also the most prone to its erosion by consequences of opportunistic control and exploitation.

An introductory section outlines descriptions of how corruption and its effects are contrary to basic needs for resilience, focussing on erosion of personal capacities and abilities; its significance to poverty and development within less-developed countries being indicated. Detailed analysis of social prerequisites for resilience is described with reference to internationally adopted definitions as a basis for discussion of their interpretation and comparison, both historic and recent. Some worldwide corrupt practices and attitudes to them are described in contexts of resilience theory, its reality and its consequences. Discussion of economic and social consequences of corruption is based upon Transparency International definitions and their shortcomings. Conclusions highlight a relationship between corruption, poverty and their impacts of natural hazards and causes of disasters. Depletion of national incomes by corruption relates to causes of poverty and the need for removal of corrupt practices at all social levels. Improved quality of life may then permit emergence of required prerequisites for resilience.

## Introduction

Investigated and published more often as a financial issue (e.g. Drury et al. 2006; Klein 2007; Transparency International 2016a, 2016b; Zucman 2015), corruption in its various guises imposes wide-ranging social consequences, especially when established long-term to the extent of having become ‘normal’ and when its networks, influences and consequences reach community and domestic contexts.

Corruption is a cause of low development (Zucman 2015:34–55) and exacerbates poverty where poverty prevails; corruption, therefore, needs to be included amongst causes of the consequences of poverty, such as debt, incapacity, mental despair and despondency (Ray 1986). Within influences as powerful as poverty, corrupt practices, in many forms and over long periods of time, may affect all and every exchange or transaction at every level of society, imposing additional insidious and negative influences upon the emergence of resilience. With little or no hard evidence for outsiders and rarely spoken of to those it does not dominate, in its numerous forms, the invisible, outwardly imperceptible practices of corruption are a cause of debilitating, pervasive and penetrating impacts upon day to day behaviours, ways of life and of well-being (Chabal & Daloz 1999; Hartmann & Boyce 1990; Hoogvelt 1976; Lewis 2008b, 2011b, 2011c; Ray 1986).

Whatever resource and effort may be introduced for its purpose, resilience may be impeded, or may not materialise, where indigenous systems of control prevail and where social capacities are consequently inadequate.

Prevailing incapacities may have been caused by a variety of circumstances, such as: long-term political repression (Lewis 2013a), ill-considered occupation or re-occupation of hazardous and damaged locations (Lewis 2013b), direct experiences of catastrophe, deaths, injury, shock or other consequences, or long-term poverty of a degree to so seriously deplete initiative and well-being as to induce physical and mental inertia (Symons 1839). Poverty is commonly assumed to be because of a country being poor whilst, in reality, poverty exists in most societies (Lewis & Lewis 2014). Any or all of these consequences may have been, or may yet be, experienced over long periods of time, separately or simultaneously, repeatedly or continuously.

For the emergence and organisation of resilience in any context, prerequisites of individual capacity and ability (United Nations: International Strategy for Disaster Reduction [UN/ISDR] 2009) are identified as being necessary. Without capacity and without individual qualities ‘to reduce negative consequences’ of disasters and for application to ‘long-term strategies for societal change’ (UN/ISDR 2009), it is difficult to envisage how community and organisational resilience could gestate, emerge or formulate. In any context and at any level, if individuals are not resilient then how would community resilience come to prevail?

Many less developed countries are internally perceived as most corrupt (Transparency International 2016b) and some of the most corrupt are amongst those most vulnerable to natural hazards; Bangladesh, Nepal and the Philippines, for example. For the 20-year period 1996–2015, almost half of all deaths because of all natural hazards occurred in low-income countries (Centre for Research on the Epidemiology of Disasters [CRED]/United Nations International Strategy for Disaster Reduction [UNISDR] 2016a). In contexts such as these, it is pertinent to ask: how much is a country’s apparent poverty because of corruption in governance and commercial mismanagement, and how many basic components of resilience, such as well-being, capacity and ability could have, indeed should have, been induced and supported in the name of indigenous normal good governance and social development?

## Social prerequisites for resilience

Resilience has been defined as:

The ability of a system, community or society exposed to hazards to resist, absorb, accommodate and recover from the effects of a hazard in a timely and efficient manner, including through the preservation and restoration of its essential basic structures and functions. (UN/ISDR 2009:n.p.)

A definition which may be read either as an assumption that ability exists or as a caution that it may not (Lewis 2013a).

Resilience theory originated in ‘late 20th century American cities’ (Davoudi et al. 2017), in which ‘radical self-sufficiency’, autonomy and ‘self-dependence’ are facts of life for all but the poorest (Lewis 2013a). What is not known is what kind of ‘community or society’, or what personal, local and national resources, were assumed as the basis of its definition.

Nonetheless, requirements for resilience have come to assume a universal capability of people to absorb stress and to transform and adapt to managing risks. In short, to deal with crises and disasters, people’s capacity being dependent upon demographic, social, cultural, economic and political factors which may vary. Resilient societies are expected to be able to overcome the impact upon them of natural hazards ‘either through maintaining their pre-disaster social fabric, or through accepting marginal or larger change in order to survive’ (UN/ISDR 2009:n.p.). Required is the capacity to adapt ability in the creation of capability for recovery (Wisner 2016). Thus, the concept of resilience is linked to the concept of change (Manyena 2006) which may be technological, economic, behavioural, social, cultural (Gaillard 2007) or political (Lewis 2013a), but in conditions of pervasive poverty, there may not be the ability to ‘accommodate and recover’, or for ‘maintenance of social fabric’; least of all the ability, capacity and capability to ‘change in order to survive’ and ‘in a timely and efficient manner’ (UN/ISDR 2009:n.p.).

The UN/ISDR definition goes further in recognising that resilience ‘is determined by the degree to which the community has the necessary resources and is capable of organising itself both prior to and during times of need’ (UN/ISDR 2009:n.p.). Consequently, ‘resilience’, once a characteristic of individuals, has come to be widely applied to preventive motivations as well as to post-disaster contexts, and to being relevant to drought, flood, climate, infrastructure, industrial complexes, businesses, cities, communities and administrations and governments and their politically stated objectives (e.g. Resilience-Scan 2016). Poverty and resilience cannot be *assumed* to go together (Boubacar et al. 2017); moreover, in realities of the aftermath of any catastrophe, whether or not in conditions of prevailing poverty, is it not more than likely that ‘ability’ may be severely depleted or may not exist at all (Lewis 2013a)? Resilience theory is said to risk becoming ‘another carrier of neoliberal ideologies, politics and practices with negative implications for social justice and democracy’ (Davoudi et al. 2017:n.p.).

External initiatives applied as preliminaries towards achievement of community resilience over time, for example, by the improvement of living conditions, healthcare and education as described in detail from Bangladesh (Ahmed et al. 2016), may assist contexts of socially comprehensive resilience in the short-term. Focus, however, on localised and current conditions may obscure suspected but hidden causes of those conditions and the consequent need for their cessation and prevention; they may be direct or indirect consequences of questionable influences or of corrupt governance nationally and locally (Lewis & Kelman 2012).

Notwithstanding inculcation prior to crises to achieve the social capacity resilience requires, capacity may be annihilated or severely depleted in ensuing catastrophe and its aftermath. Despondency, not resilience, may become the reality, expressing not ability but inert disability. Resilience may theoretically pre-exist as a basic human quality but cannot be *assumed* to prevail regardless of realities of physical, mental and psychological incapacities, especially in contexts of poverty.

Present in any society at any time (Lewis & Lewis 2014), an early analysis of poverty in Scotland, France, Belgium, Austria and Switzerland (Symons 1839:147–148) realised that poverty has ‘… the same effect on the mind that drunkenness has upon the body’ and that poverty was:

… a main instrument in the debasement of mankind … It is not only the parent of ignorance, but it is the greater barrier to enlightenment. When a man’s whole faculties are strained to the utmost from sunrise to sunset to procure a miserable subsistence, he has neither the leisure, aptitude nor desire for information … (pp. 147–148)

It could be assumed from this description that the sufferer would not have had capacity for resilience.

Fifty-three years later, Friedrich Engels ([1892] 2009) wrote of England:

Everything that the proletarian can do to improve his position is but a drop in the ocean compared with the floods of varying chances to which he is exposed, over which he has not the slightest control. He is the passive subject of all possible combinations of circumstances … (p. 144)

It may be impractical to assume resilience where, for example, many populations are striven by conflict and warfare, millions of people are on the move as refugees and migrants, where millions more are in abject poverty and more directly where people are immediate and longer-term victims of catastrophe. Peace and stability may have been achieved in the aftermath of similar experiences but populations may have been left in fear of recurrence, a fear not conducive to the emergence of ability (Lewis 2013a; Lewis, Kelman & Lewis 2011e) and a condition which may last for many years.

Whilst communities may be, or may become resilient, they may continue to be vulnerable and at high risk (Sudmeier-Rieux 2014), continuingly prevalent causes of their vulnerability (Lewis & Kelman 2010) having been bypassed and disregarded by priorities for achieving resilience. Whereas destruction and damage are described in terms of physical impacts, these may transfer as mental, emotional, social and economic impacts upon individuals and communities. For some time, primary resources of resilience, such as capabilities of creativity, energy and leadership, may therefore be scarce commodities. Resilience anywhere will be dependent upon conditions that prevailed before disaster as well as those created by it and upon programmes for development responsive to potential contingencies of environmental hazards and disasters (Lewis 2013b). Prescribed characteristics of resilience rarely refer to preceding contexts (e.g. Twigg 2007), some least positive contexts being described by Lewis and Kelman (2012).

Resilience may not, therefore, emerge ‘on demand’, commensurately comparable with the origins of catastrophe from whatever source. This would require a different kind of resilience, not on-the-spot reactions to chaos but one that recognises resilience as a long-term process more compatibly aware of political, social and economic causative processes of inequality, vulnerability and poverty (e.g. Lewis 2013a), of which the social, as well as economic, consequences of corruption and its associated practices are a significant cause.

But stable, equable, fair and considerate communities and their regional and national administrations are a rarity; poverty, expressed according to a country’s median income, exists virtually in all countries as, in its varying degrees and practices, does corruption (Transparency International 2016b). Where politicians appear to be in power to facilitate their own incomes and lax administrative systems facilitate them to do so, corruption becomes a cause of poverty, a major impediment to equality and the ‘worm-in-the-bud’ of resilience.

## Some economic and social consequences of corruption

Corruption, as ‘the abuse of entrusted power for private gain’, has been classified as ‘grand’, ‘political’ and ‘petty’, depending on the amounts of money lost and the sector of governance in which it occurs (Transparency International 2016a). International scales of corruption, reviewed annually, are based upon *internal* perceptions of corruption as it is indigenously observed and experienced, a methodology by which it is not possible to compare one perception with another or to know how they were arrived at. This means that whilst corrupt behaviours of politicians or large corporations are reported by the media, they may or may not influence those perceptions upon which international comparisons are based. The international definitions and comparisons by Transparency International are nevertheless a principal comparative scale of corruption and its definitions.

Most corrupt practices operate on, or create, a hierarchical scale of trading, a system that ensures that costs to top-level payers of bribes may be expected to be reimbursed by the receipt of bribes from others, those lower on the scale being recipients of backhanders to them for favours given. Payments would be expected and reimbursed similarly downwards to scales of petty corruption. That socially lowest payers have no-one upon which to claim is how millions of people find themselves in endless poverty – beholden and indebted victims for further exploitation by those richer and more powerful, at whatever level, than themselves. The poor become poorer to the advantage of the rich and poverty and inequality are perpetuated. Realities of corrupt practices upon those already in poverty cannot simply be classed as ‘petty’.

Of Africa, Chabal and Daloz (1999) argue in support of corruption being ‘the norm … constituting a substantial resource’ (Chabal & Daloz:xxi), taking the view that there has always existed a wide range of activities, inclusive of corruption, which, although illicit from a strictly constitutional or legal point of view, have been regarded as legitimate by the bulk of population (Chabal & Daloz:79). They emphasise however that corruption affects all social strata ‘from billionaires to the lowliest functionary’. Consequently, dichotomy between ‘high’ and ‘low’ or ‘small-’ and ‘large-’ scale corruption is not a determinant factor; neither are differences between financial malpractice, illegal commissions, small graft, open abuse of power, and petty pilfering. Nor do these authors believe some forms of corruption are more reprehensible than others, all forms of corruption being part of an interconnected whole (Chabal & Daloz:98).

Others (e.g. Hoogvelt 1976) see corruption as ‘the only means of integrating marginal groups into a disjointed social system’ (Hoogvelt 1976:132) but where that is the case, corruption should not be allowed to be a licence for social injustice by forcefully keeping in power undeserving elites (Hoogvelt 1976:137).

*Grand corruption* in governments’ higher echelons (Transparency International 2016a), necessarily filters down, with its consequences, throughout all functions of all societies. Politicians and commercial operators, privately and corruptly, are known to have siphoned collectively enormous amounts of money, much of it from development funding, often from their own disaster-prone countries and very often into private bank accounts in the countries that were the origin of the aid (Ndikumana & Boyce 2011).

Known as ‘illicit financial flows’ and merged with corruption because of their secrecy, tax evasion and avoidance, and with sources possibly related to more strictly defined corruption, dishonest transactions on a huge scale (Zucman 2015:34–55) have emerged as evidence of why some countries have remained ‘less-developed’. Money, illicitly taken from external funding intended for development purposes, is a likely cause of reduced domestic investment in basic needs of housing, sanitation, health and education, an explanation of why poverty has prevailed as the principal cause of vulnerability (Lewis 2015) and its associated disaster losses and social incapacities, and why such issues have not been matters of development priority by some national governments and indigenous organisations.

A report from the Philippines (Rey 2016) concludes that, from 1960 to 2011, approximately $410.5 billion left that country in ‘illicit financial flows’; a figure stated as being 154 times the national budget for health, 52 times that for social protection, 39 times that for education and 25 times that for infrastructure for the same period.

The overall cost to developing countries between 2000 and 2008, of corruption and trade mispricing (trade as a vehicle of monetary transfer), was approximately $6.5 trillion (Kar & Curcio 2011), a subsequent United Nations Development Programme (UNDP) report indicating that $197bn, a significant share, had accrued from those countries categorised as least developed (UNDP 2011). A more recent report describes illicit financial flows from eight countries, including Bangladesh and Nepal, as a symptom of poor governance and dysfunctional regulation, and having the following consequences:

- undermining of domestic resource mobilisation by eroding the tax base
- causing greater dependency on official development assistance
- reducing domestic investment and slowing poverty reduction efforts and worsening of inequality (UNDP 2014).

Illicit financial flows from developing countries worldwide in 2013 totalled $1.1 trillion, a figure greater than the combined total of foreign direct investment and net official development assistance received by those economies in that year. As examples, illicit financial flows between 2004 and 2013 from Bangladesh totalled $5588 million, from Nepal $567m, and from the Philippines $9025m (Kar & Spanjers 2015).

*Political corruption* is the manipulation of policies, institutions and rules of procedure in the allocation of resources and financing by decision makers, who abuse their position to sustain their power, status and wealth (Transparency International 2016a).

An investigation in Bangladesh of self-reported compliance with corporate governance, examined enforcement documents of the Securities and Exchange Commission against actual corporate governance compliance from 2007 to 2011 (Nurunnabi, Hossain & Al-Mosa 2016). The authors observe that corruption and lack of enforcement in Bangladesh induced falsification of formal financial reporting under both democratic and military governments (2007–2008). The extent of falsification of information is stated as a cause of alarm for both local and international policy-makers and local and international investors. One thousand one hundred and ninety-four Bangladesh Securities and Exchange Commission’s enforcement documents were evaluated and 20 semi-structured interviews were conducted.

In 2007, the government of China had more than 1200 laws, rules and directives against corruption, but implementation was ineffective. With only a 3% likelihood of a corrupt official being sent to jail, corruption was a low-risk high-return activity. Even low-level officials had the opportunity to amass an illicit fortune of tens of millions of yuan. The secretary to the Chinese Communist Party in Janwei county of Sechuan province acquired 34 million yuan (£3 467 952/$5 096 000) and the colleague of another Chinese Communist Party (CCP) secretary, his city’s anti-corruption chief, collected bribes worth more than 30 million yuan (£346 794 000/$4 497 000; Lewis 2008b).

Corruption in China is concentrated in those sectors with extensive state involvement, such as infrastructure projects and government procurement, the consequent increased costs of which, during a 10-year period, were estimated as 10% of spending (ending in 2005). Such a depletion of funds contributed to environmental degradation, social instability and inadequate health care, housing and education:

To estimate roughly the direct costs of corruption, we can suppose that ten per cent of government spending, contracts, and transactions is used as kickbacks and bribes or is simply stolen. (Lewis 2008b:n.p.; Pei 2007:n.p.)

In relative terms, developing countries are the most affected by volumes of wealth held abroad, calculated for 2014 as 30% for those of Africa (Zucman 2015:53). But between 1970 and 2008, an examination of capital sent from 33 African countries concluded that over that 38-year period, ‘capital flight’ amounted to $735bn, a sum roughly equal to 80% of the combined GDP of those countries during that time. The period of this study indicated that the sum involved was ‘not a transitory product of unusual circumstances but rather an outcome of persistent underlying causes’ (Ndikumana & Boyce 2011:46). An earlier study by the same authors concluded that this sum was ‘from assets belonging to a narrow, relatively wealthy stratum of populations while, in consequence, public external debts are born by the people through their governments’ (Ndikumana & Boyce 2008 quoted in Shaxson 2011:158). Similar procedures making use of offshore tax-havens have operated on behalf of the rich and at the cost of the poor within many countries (Shaxson 2011). Overall, by its hierarchy of bribery and graft, corruption for the benefit of the few means continued and exacerbated poverty for the many and simultaneous breakdown or malfunction of hospitals, clinics and health care (Ndikumana & Boyce 2011:74–83, cited in Lewis 2015). Corrupt practices are widespread within entire commercial sectors of some countries, and are known to have been causes of serious inadequacies such as building failure (Ambraseys & Bilham 2011; Lewis 2005, 2008a, 2008b).

Political elites of some developing countries are known to accumulate capital because of the fragility of their position and constant threat to their political survival (Hoogvelt 1976:137); partly for that reason, large sums are transferred to safer European accounts or to the many global ‘tax-havens’ (Shaxson 2011).

In a large scale public works contract in Italy, endemic collusion between levels of administration, elected officials, bureaucrats and private contractors made it obvious that for such abuse of public office for personal gain to persist countrywide, elected officials are necessarily and regularly involved. Extensive and persistent corruption in any sector, could not be regarded as a phenomenon isolated from its broader political context; a political environment of corruption involves a non-benevolent principal rather than being a benign bureaucratic or institutional slippage from a benevolent one (Golden & Picci 2005).

*Petty corruption* refers to everyday abuse of entrusted power by low- and mid-level public officials in their interactions with ordinary citizens, when seeking to access basic goods or services in hospitals, schools, police departments and other agencies (Transparency International 2016a). These corrupt practices are rarely spoken of and expectations of bribes are rarely applicable to anyone not known to the locality. Without long-term presence and discrete research (e.g. Hartmann & Boyce 1990; Ray 1986), assured evidence of ‘petty’ corruption remains obscure.

In 2013, a Philippines national survey (Office of the Ombudsman 2014) indicated fewer families to have given bribes or ‘grease money’ in 2013 than in 2010. The survey found that more people in ‘the lower income stratum’ were more likely to pay bribes or ‘grease money’ despite ‘their lower financial capacity’, assumed by the report as to ensure government social services essential to them were made available.

Of West Africa, Hoogvelt (1976), believes corruption affects everyone:

patients offering bribes to nurses in hospital to persuade them to pass on a bed-pan; traffic offenders bribing police officers to waive the fine; tax collectors adding their personal increment to inland revenue extractions; councillors awarding contracts to firms in which they (or their kin) have a financial stake; educational officers giving government scholarships to their cousins; and political candidates buying the votes of entire electoral districts. (pp. 128–129)

Hoogvelt adds that corruption at the law enforcement level, involving lower echelons of civil and public services, is where contact between administrations and the public are most frequent and where, therefore, the greatest volume of corruption occurs – though the amount of damage done and money involved may well be greater at higher levels (Hoogvelt 1976:130).

Corruption retains society’s levels in place, corrupt behaviours at lower social levels being a microcosm of those at upper levels. Where larger landowners control most land of their district, consequences at lower levels impact upon minor landholders, share croppers and labourers who own little or no land (Hartmann & Boyce 1990:7); a system that ensures those at each social level will remain at that level, the rich as well as the poor, and the poor will remain beholden to, and controlled by, the rich.

An exception from Bangladesh illustrates the generality: Mahmud was a poor student living in Johir Ali’s house and tutoring his children in exchange for a room and board. After Mahmud graduated from secondary school, Johir Ali is said to have paid a 500 *taka* bribe to secure him a job as a *tahsildar*, a government land-tax officer who records the amount of land which tenants held on lease. Tenants were often in arrears with their rent payments and at risk of dispossession, providing Mahmud opportunities to help himself by helping others with their payments. Mahmud charged a fee for his services, but since it was less than the going price of land, most tenants were happy to comply. During his years as a tax officer, Mahmud accumulated considerable capital, which he invested in land and later left his government job to take up the management of his sizeable holdings (Hartmann & Boyce 1990:55).

Every service demands a kickback or backhander additional to any legal payment that may be required. Larger landholders in line for development aid, such as for the drilling of wells, will buy up numbers of offers to which they may be eligible to sell on to lesser landholders either as wells or as water from wells in their ownership. Larger land owners control lesser landholders and smaller crop growers. From development aid, the poor get temporary employment (e.g. from the use of water), the rich reaping repeated capital gains from the installation of a well (Hartmann & Boyce 1990:257, 262, 272, 274).

As a consequence of ‘tremendous power’ wielded in Bangladesh by the rural rich, ubiquitous corruption pervades every sector at every level and is stated as being a principal hindrance to the achievement of self-reliance by the rural poor. Wealthy landowners, physicians, shopkeepers, chairmen or members of the *union* *parishad* (local government), have long-lasting connections and alliances with government in the capital, officials of all ranks, lawyers, judges and powerful politicians. Sustained by bribes, gifts, marriage and birth, these alliances, enable the rural rich to safeguard their narrow self-interest, ‘committing crimes if necessary and getting away’ (Ray 1986:24–25).

A study in flash flood prone north-eastern Bangladesh (Choudhury & Haque 2016) identifies social power structures, imposed by local political and commercial elites, as serving to diminish local adaptive capacities and consequently as an impediment upon resilience. Petty corruption, in the form of bribery referred to in the study, emerges as an understated but consistent component of impositions upon those in poverty; expressed as the eponymous quotation: ‘We are more scared of power elites than the floods’.

Reports of occasional local optimism (e.g. Hossain 2016) need to be set against realities of corruption at all relevant levels and scales (Transparency International 2016b) and of its social consequences.

## Conclusion

Contexts of poverty may be created by corrupt practices at higher levels of government and commercial management (Transparency International 2016a; UNDP 2014), exacerbated and perpetuated by social systems imposed upon people and their communities for purposes of domination and exploitation to facilitate ‘petty’ corrupt practices (Choudhury & Haque 2016; Hartmann & Boyce 1990; Ray 1986).

Contexts of poverty are known to be amongst the most vulnerable and the most disaster-prone (Lewis & Kelman 2012). Of countries lowest on the internally perceived international corruption scale (Transparency International 2016b), several are amongst the poorest developing countries (Ambraseys & Bilham 2011). Of the 168 countries on the scale, Myanmar is 147th, Bangladesh is 139th, Nepal is 130th and the Philippines is 95th; of low-income and lower middle-income countries (CRED/UNISDR 2016b), Myanmar is 147th and Pakistan is 117th. Whilst consistent correlation between corruption and disaster impacts is unlikely, disaster mortality is highest in Haiti, at 158th amongst the lowest on the corruption scale and highest for disaster mortality.

In these and numerous other countries, poverty persists for large numbers of people caused to be at risk by pernicious political, commercial and social realities which result from discrimination and displacement, impoverishment by others’ self-seeking expenditure, denial of access to resources, and corrupt siphoning of public money that may be otherwise spent to the public good (Lewis & Kelman 2012); sub-cultures working to favour the few but in opposition to the interests of the many (Lewis 2015).

As a perpetrator and perpetuator of poverty and inequality (Alexander 2016; Lewis 2011a; Lewis & Kelman 2012), by its various guises and their consequences, corruption is a ubiquitous impediment of abilities to ‘accommodate and recover’, and ‘change in order to survive’, the basic functions of resilience (UN/ISDR 2009).

Further, where aspects of national income are diverted to private accounts and payments of bribes are set against declared company profits, the basis upon which national tax incomes are formed is reduced. Income which could have been spent for the benefit of society at large is depleted on such a scale that housing, education, sanitation, nutrition and healthcare (Ndikumana & Boyce 2011), for example, are threatened or rendered inadequate (Lewis 2011a). Corrupt behaviours leading to depletion of national and local incomes are an explanation for why works for basic community development are perceived as necessary for preliminary projects to precede projected inputs for sustainability and resilience (e.g. Ahmed et al. 2016).

Until corrupt practices are traced and stopped, it may not be realistic to expect villagers in long-term poverty to turn to new activities merely by advising them to do so: ‘After all, decades of abject poverty has instilled in them a deep fear that trying anything new may be disastrous’ (Ray 1986:4). Traditionally ingrained corrupt practices may seem inseparable from social norms, the introduction of new practices being seemingly ‘next to impossible’, however essential they may be for longer-term social development to succeed.

Only ‘rugged common sense’ enables the poor to survive decades of exploitation by a ruling urban elite. Famished villagers cannot work towards change to the system by which they are oppressed unless they have achieved a minimum of nutrition and physical strength, ill health being inextricably linked to illiteracy, malnutrition, superstition, unemployment and agricultural backwardness (Ray 1986:vii–viii, 3–4) – a close comparison with statements made by Symons (1839) with reference to Edinburgh and European capitals.

Corruption is not only a financial issue; corruption creates social systems compliant to its practices and influences entire societies and the social relationships they contain. In these circumstances and where systemic corruption persists, attempts to induce and to inculcate resilience to hazards and crises, if successful in any short-term, may be unlikely to succeed in any longer-term.

The start of any programme for rural resilience has to be the depletion of those ‘traditionally ingrained corrupt practices’. If famished villagers who have not achieved a minimum of nutrition and physical strength, cannot work towards change to the system by which they are oppressed, then externally applied programmes for purposes of creating resilience are unlikely to succeed in any longer-term. Corruption and its consequences will make any kind of social development programme unsustainable and community resilience is unlikely until individual resilience amongst individuals is itself sustainable (Lewis 2015).

Repeatedly, necessary injections of programmes and projects for sustainability and resilience might suggest their temporary presence to be due not to unavailable financial resources but to indigenous illicit misappropriations of financial capital. Corruption denies and impedes personal and community empowerment for change, the basic requirement for disaster risk reduction (Von Meding & Forino 2016). How much less vulnerable, and how much more resilient would populations be, without social impediments and financial draining at all levels imposed by corruption in any and all its guises?

Development programmes of wider inclusivity are emerging from responses to climate change and its consequences (Ahmed et al. 2016; Kelman et al. 2016; Lewis 1999). Adjustments for this wider inclusivity could be made to go further and to incorporate measures for annihilation and prevention of corrupt practices which, with poverty reduction, disaster risk reduction and resilience, would be an inclusivity serving to ensure improved long-term developmental effectiveness, sustainability and reliability.

Social consequences of corruption have been examined and considered and found to be negatively influenced against the required prerequisites for resilience. A question that remains is not ‘can resilience exist in contexts of corruption?’ but rather, ‘would the inducement of resilience be less necessary in non-corrupt contexts?’
